# Highly specific *in vivo* gene delivery for p53-mediated apoptosis and genetic photodynamic therapies of tumour

**DOI:** 10.1038/ncomms7456

**Published:** 2015-03-05

**Authors:** S.-Ja Tseng, Zi-Xian Liao, Shih-Han Kao, Yi-Fang Zeng, Kuo-Yen Huang, Hsin-Jung Li, Chung-Lin Yang, Yu-Fan Deng, Chi-Feng Huang, Shuenn-Chen Yang, Pan-Chyr Yang, Ivan M. Kempson

**Affiliations:** 1 Department of Internal Medicine, National Taiwan University College of Medicine, Taipei 10051, Taiwan; 2 Institute of Medical Science and Technology, National Sun Yat-sen University, Kaohsiung 80424, Taiwan; 3 Research Center for Tumor Medical Science, China Medical University, Taichung 40402, Taiwan; 4 Institute of Biotechnology, National Taiwan University, Taipei 10617, Taiwan; 5 Graduate Institute of Life Sciences, National Defense Medical Center, Taipei 11490, Taiwan; 6 Departments of Neurosurgery, Chang Gung Memorial Hospital, Taoyuan 33305, Taiwan; 7 Department of Industrial and System Engineering, Chung Yuan Christian University, Taoyuan 32023, Taiwan; 8 Institute of Physics, Academia Sinica, Taipei 11529, Taiwan; 9 Institute of Biomedical Sciences, Academia Sinica, Taipei 11529, Taiwan; 10 NTU Center for Genomic Medicine, National Taiwan University, Taipei 10051, Taiwan; 11 Ian Wark Research Institute, University of South Australia, Mawson Lakes, South Australia 5095, Australia

## Abstract

Anticancer therapies are often compromised by nonspecific effects and challenged by tumour environments’ inherent physicochemical and biological characteristics. Often, therapeutic effect can be increased by addressing multiple parameters simultaneously. Here we report on exploiting extravasation due to inherent vascular leakiness for the delivery of a pH-sensitive polymer carrier. Tumours’ acidic microenvironment instigates a charge reversal that promotes cellular internalization where endosomes destabilize and gene delivery is achieved. We assess our carrier with an aggressive non-small cell lung carcinoma (NSCLC) *in vivo* model and achieve >30% transfection efficiency via systemic delivery. Rejuvenation of the p53 apoptotic pathway as well as expression of KillerRed protein for sensitization in photodynamic therapy (PDT) is accomplished. A single administration greatly suppresses tumour growth and extends median animal survival from 28 days in control subjects to 68 days. The carrier has capacity for multiple payloads for greater therapeutic response where inter-individual variability can compromise efficacy.

## Abstract (web summary)

Alterations of p53 are associated with more than half of all human cancers. Here the authors present a new pH-sensitive nanoparticle that is delivered via systemic circulation and combines gene delivery to restore p53 with expression of Killerred protein to induce photosensitization.

## Graphical abstract

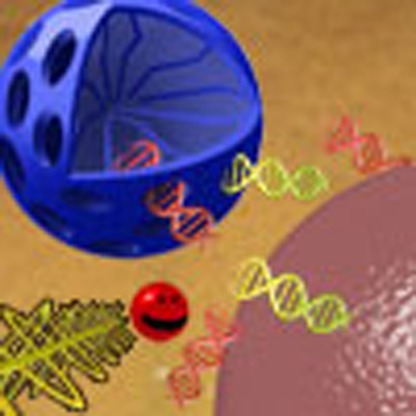

The mutation and dysfunction of protein 53 (p53) has been associated with over 50% of human cancers^1^. Mutant p53 not only loses tumour suppressive abilities^2^, but additionally leads to promotion of tumorigenesis^3^. Replacement of the genetic aberrance in cancer cells is one of the most promising therapeutic approaches^4^,^5^,^6^,^7^. It is clear that p53 has great significance in cancer growth and therapy. In a specific example, p53 suppressed cancer invasion by prompting the degradation of Slug protein, an invasion promoter in patients with non-small cell lung cancer (NSCLC)^8^. Mutant p53 compromises Slug degradation, resulting in Slug accumulation and cancer invasiveness. In addition, cellular generation of reactive oxygen species (ROS) can act either upstream or downstream of p53-mediated apoptosis^9^. Studies have shown that ROS increases c-myc, which inhibits cell cycle arrest but does not influence p53-transactivated pro-apoptosis gene, PUMA^10^. ROS can also enhance the post-translational modification of p53, such as phosphorylation and acetylation, both of which lead to p53 protein stabilization and activation^11^. On the contrary, the overexpression of p53 transactivates redox active proteins, that is, quinone oxidoreductase (NQO1; ref. 12) and proline oxidase (POX)^13^ and their elevation results in oxidative stress and subsequent cell death. These pieces of evidence show that ROS and p53 pathways are intertwined; excessive amounts of either pathway may augment the other, consequently leading to cell apoptosis. These avenues in the treatment of cancer can be approached with the development of effective gene delivery. Polymeric-based carriers offer great promise, however, they generally fail in attempts to translate *in vitro* effect to *in vivo* models^14^. Chemotherapy is one major approach for treating NSCLC; however, progression-free survival rates depend significantly on the individuals’ chemotherapy response and extent of tumour metastasis. The genetically-encoded red fluorescent protein (KillerRed) leads to irreversible DNA damage and cell killing via an apoptotic pathway upon irradiation with a narrow light bandwidth of 520–590 nm due to formation of highly-cytotoxic levels of ROS^15^,^16^; offering additional options in treatment of tumour and study of tumour radiosensitization. Most current photodynamic therapy (PDT) regimens are based on a single administration^17^. Thus, successful demonstration here of a carrier for replacement of the aberrant p53 gene in combination with *in situ* PDT sensitization offers an opportunity for effective gene therapy with a single administration.

Numerous approaches are being taken to reconstitute wild-type p53 expression in tumour and to use the function of the p53 pathway as a reliable predictor of therapy response and patient prognosis^4^,^5^,^6^,^7^. Approved studies in the United States implemented p53 gene-based therapies using viral vector delivery, in various clinical settings^6^,^7^. Side effects were mild but the treatments required local injection into the tumour site and may not be effective in all patients compared with current best standard of care, especially after metastasis. Attempts to improve the efficacy by modifying p53 and enhancing delivery remain challenging. Among various polymeric carriers available, polyethylenimine (PEI) was utilized to transfer genes for *in vitro* and *in vivo* transfection^18^. Modified PEI has been found to be reasonably biocompatible^18^. Alternatively, the incorporation of negatively charged polymer into the original polymeric formulation has also enhanced the cellular uptake and transfection efficiency (TE)^16^,^19^. However, a challenge in implementation concerns its lack of specificity in targeting tumour and cellular uptake from systemic administration.

pH-sensitive activation and delivery is proving to be an attractive trigger for greater efficacy in treatment of tumour. Particular attention targets pH-responsive carriers/materials exploiting acidic microenvironments^20^,^21^,^22^. In solid tumour, accumulation of lactic acid leads to slight lowering of extracellular pH (~pH 6.7–7.0; ref. 23). The low tumour pH primarily results from a high rate of glycolysis, which can produce acids under both aerobic and anaerobic conditions. We previously developed highly pH-sensitive complexes of 150–250-nm diameter carriers and hydrogels for controllable transfection^24^ or extracellular delivery^20^,^22^,^25^. Here we present an improved technique that switches to promote cellular uptake and delivery of plasmid DNA of p53 and KillerRed triggered by tumour acidosis *in vivo* (Fig. 1a). Negatively charged DNA-complexes were formed with plasmid DNAs, branched PEI (bPEI_25K_, Mw=25,000) and poly (poly(ethylene glycol; PEG-His-PEG-Glu) consisting of tuned ratios of PEG, histidine (His) and glutamic acid (Glu) (Supplementary Fig. 1). In this work we make a comparison between this carrier, which we term as the DNA-complex (with tuned pH response), with a bPEI25k/DNA-complex (which lacks the PEG and amino acid residues and resulting pH sensitivity). Exposure of the DNA-complex to acidic pH (pH=6.8) triggers protonation of the amino groups and an *in situ* charge reversal, leading to cellular uptake and transfection. The zeta potential of the DNA-complex recovers once the environment returns from acid to neutral pH. Such a carrier is highly versatile in its loading as well as effective in being responsive to the ‘targeted’ tumour tissue microenvironment from systemic administration. In the present study, we simultaneously rejuvenate the p53-mediated apoptosis signalling pathway and enable photoinduced ROS generation; effectively suppressing tumour growth of an aggressive human NSCLC cell line (H1299) in athymic BALB/c nude mice.

## Results

### Characterization of pH-dependent regulation

**Figure 1:**
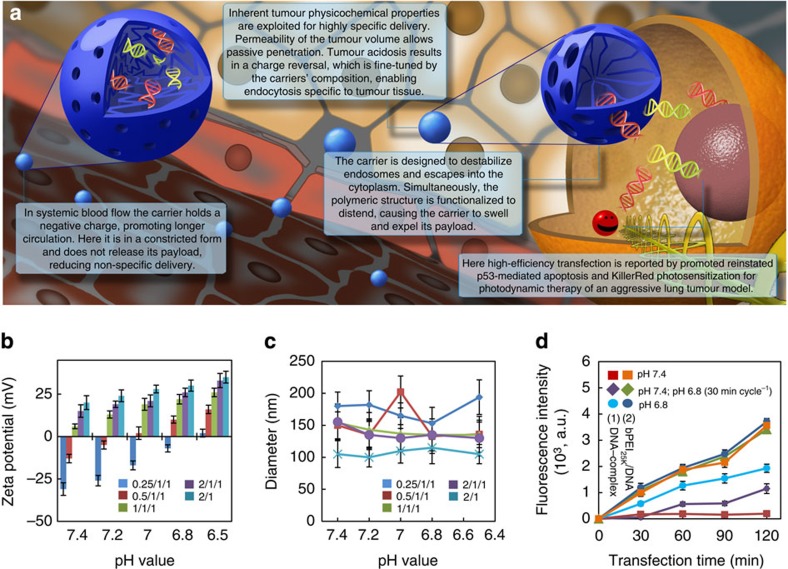
A highly pH-sensitive carrier. (**a**) A schematic representation of the gene loaded carrier. Its biocompatible, contracted, negatively charged form circulates freely in a passive form until extravasation into tumour tissue characterized by lowered pH. Tumour acidosis switches the carriers charge to enhance cellular interaction and uptake. Protonation of the carriers’ amino groups leads to distention of the polymer chains, dilation and expulsion of payloads. Here p53 and KillerRed genes transfect tumour cells. p53 triggers apoptosis while illumination of KillerRed protein generates ROS and subsequent intracellular damage, further promoting cell apoptosis. (**b**,**c**) ζ-Potential (**b**) and hydrodynamic diameter (**c**) of the DNA-complex and bPEI_25K_/DNA-complex as a function of pH for varying bPEI_25K_/DNA/copolymer weight ratios. Data show the mean of the measurements conducted in triplicate±s.d. (**d**) Fluorescence intensity after cellular uptake of the bPEI_25K_/DNA-complex (bPEI_25K_/DNA weight ratio: 2/1) or DNA-complex (bPEI_25K_/DNA/copolymer weight ratio: 0.5/1/1) monitored using Alexa Fluor 488 labelling of plasmid DNAs over the period of several cycles of pH between 7.4 and 6.8 or incubated at only pH 7.4 or 6.8. Results show mean of measurements conducted in triplicate±s.d. Oscillating charge of the carrier results in a step-like response in cellular uptake of the DNA-complex with fluctuating pH.

Zeta potential and size of DNA-complexes were measured by dynamic light scattering and the formation of the DNA-complex was verified by the changes in the surface charge and particle size at various pH values. The zeta potential varied between negative and positive values (−31±3.7 to 15±3.5 mV at pH 7.4) depending on the change of weight ratio of bPEI_25K_/DNA/poly(PEG-His_0.5_-PEG-Glu_0.5_) from 0.5/1/1 to 2/1/1 (Fig. 1b). Specifically, the extent of protonated amine groups of DNA-complexes increases with a decrease in pH from 7.4 to 6.5, which in turn, increases positive-charge density on the DNA-complex. Interestingly, the change from a negatively charged DNA-complex to positively charged could be tuned to occur when the pH value changed from 7.4 to 6.8 at 0.5/1/1 (w/w/w), indicating that the *in situ* charge reversal of DNA-complex can be achieved in an *in vivo*-relevant pH range for the tumour microenvironment. Such a change of the zeta potential augments the DNA-complex cellular interaction and consequently greater cellular uptake^24^,^25^. In various formulations of the DNA-complex’s weight ratios, the spherical DNA-complex had a diameter of *ca.* 100–200 nm over a wide range of pH (Fig. 1c and Supplementary Fig. 2). Following treatments, we examined effect of the DNA-complex and bPEI_25K_/DNA-complex on cell viability. An improved biocompatibility for lower ratios of PEI may be attributed to a more appeasing net charge (Supplementary Fig. 3)^19^,^24^,^25^. Taken together, the DNA-complex at the optimized weight ratio of 0.5/1/1 was suited to efficiently express p53 and KillerRed in tumour for localized cellular uptake and triggered functionality. Comparisons were made to the bPEI25k/DNA-complex as a control, having a 2/1 ratio, which was optimal for the maximum transfection *in vitro*.

Consequently, to evaluate the effect of pH on cellular uptake of the DNA-complex, the pCMV-p53 and pKillerRed-mem were labelled with fluorescein as a detection signal for flow cytometry. For the DNA-complex, fluorescence intensity increased when cells were incubated at pH 6.8 (Fig. 1d). After changing to pH 7.4, fluorescence intensity was maintained at a comparable level. Appreciably little fluorescence was measured for cells only exposed to pH 7.4 (in stark contrast to the bPEI_25K_/DNA-complex), indicating the necessity for pH-triggered charge reversal of the DNA-complex for cellular uptake. It is important to note that this result indicates that delivery occurs due to polymer-mediated cellular uptake dependent on the extracellular environment. In addition, the intensity of fluorescent signals of treated cells continuously increased for those exposed to the DNA-complex incubated in the slightly acidic environment (pH 6.8). Switchable release from the carrier is indicated by the data for alternating between pH 6.8 and 7.4, exemplified by the data points between 60 and 90 min when pH reverted to 7.4 (purple diamonds). Emphasis of the critical need for carrier design is presented in the data showing that for all the treatments of the bPEI_25K_/DNA-complex, the fluorescent intensities of cells in both pH conditions achieved consistently similar levels, indicating non-pH-mediated cellular uptake of bPEI_25K_/DNA-complex. Consistent with previous observations, these data indicate that the positively charged complex facilitates an association with the anionic cell membrane^24^,^25^. Exposure to fetal bovine serum (FBS) was shown, herein, to have the comparable fluorescent intensity of cellular uptake of test complexes in treated cells (Supplementary Fig. 4).

### Effect of pH on polymeric transfection of p53 and KillerRed

**Figure 2:**
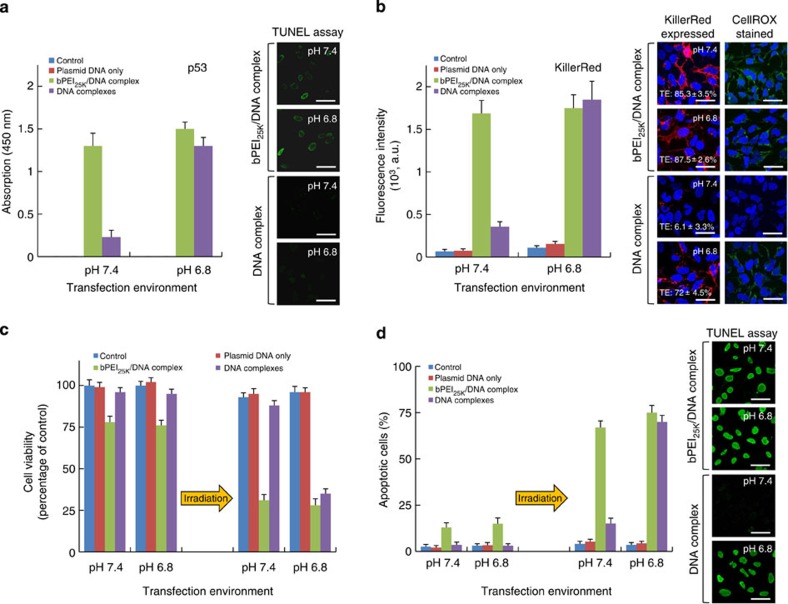
*In vitro* gene expression at pH 7.4 and 6.8 transfection. (**a**,**b**) The protein expression of p53 (**a**) and KillerRed (**b**) via DNA-complex or bPEI_25K_/DNA-complex delivery in H1299 cells in culture medium at pH 7.4 or 6.8, 48 h post transfection. p53 protein in cell lysates was measured by ELISA assay using 450 nm absorbance for quantification. Confocal images showing the p53-mediated apoptosis was observed by TUNEL assay using a fluorescein. The fluorescence intensity and TE of KillerRed-positive cells were analysed by flow cytometry. Confocal images show KillerRed expression or ROS generation mediated by KillerRed photoactiviation was observed in transfected cells, in which the CellRox green dye and DAPI stains identify ROS and nuclei, taken 20 min exposure (593 nm, 100 mW cm^−2^). (**c**) Cell viability of transgene-expressing cells incubated at pH 7.4 or 6.8 with and without irradiation with a yellow laser, determined by the MTS assay. (**d**) Percentages of apoptotic cells stained by TUNEL assay incubated after a light exposure of 20 min and different pH values, analysed by flow cytometry. Confocal images show that the apoptotic cells were observed by TUNEL assay. All results show mean of measurements conducted in triplicate±s.d. Scale bar, 50 μm.

We quantified the efficacy of p53 expression through the DNA-complex or bPEI_25K_/DNA-complex in an additional incubation of 48 h by the enzyme linked immunosorbent assay (ELISA) using the p53-deficient H1299 cells (Fig. 2a). The p53 expression observed in the DNA-complex-transfected cells increased noticeably with the acidic environment. The p53 expression with bPEI_25K_/DNA-complex had no significant difference between pH 7.4 or 6.8, evidencing the lack of pH-responsive uptake of the bPEI_25K_/DNA-complex. In the treatment with DNA-complex at pH 7.4, no labelling resulting from apoptosis signalling indicated by TUNEL (terminal deoxynucleotidyl transferase (TdT) dUTP nick-end labelling) assay was observed, implying that the positive immunocytochemical response was not caused by endogenous cell peroxidases. However, bPEI_25K_ can induce dose-dependent damage to cell membranes and subsequently leads to the initiation of apoptosis due to a high charge density, indicated by the cytotoxic effect for the bPEI_25K_/DNA-complex^26^.

**Figure 3:**
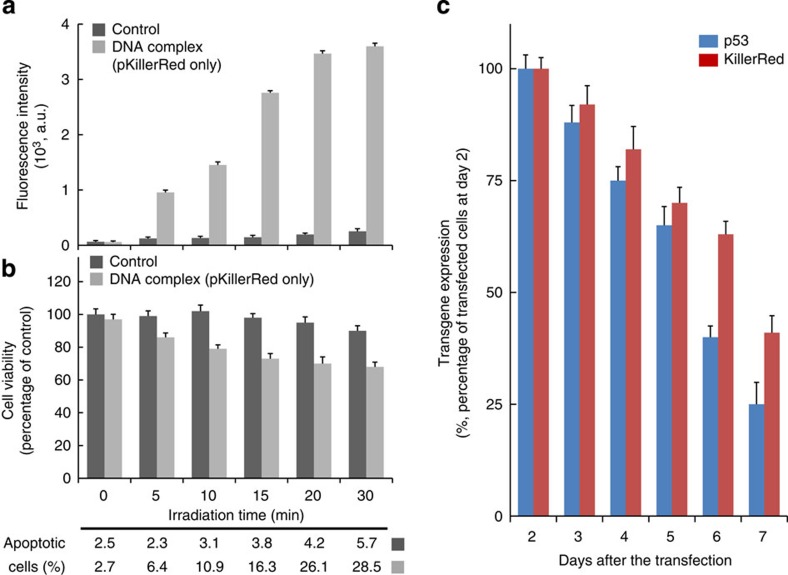
KillerRed photoactivation and degradable transgene expression. (**a**) Fluorescence intensity of ROS generation by KillerRed-positive cells incubated at different light exposure time periods (593 nm, 100 mW cm^−2^), analysed by flow cytometry. The cells were transfected with DNA-complexes containing only plasmid DNA of KillerRed at pH 6.8. Results show the mean of the measurements conducted in triplicate±s.d. (**b**) Results of the viability and apoptosis of KillerRed-positive cells incubated at light exposure time points determined by the MTS assay and TUNEL assay. Results show the mean of the measurements conducted in triplicate±s.d. The untreated cells were used as a control. (**c**) Kinetics of gene expression in cells transfected with the DNA-complex (weight ratio of 0.5/1/1) at pH 6.8. Results show mean of measurements conducted in triplicate±s.d.

In addition, flow cytometry quantified fluorescence intensity and TE of KillerRed-positive cells. The bPEI_25K_/DNA-complex showed high intensities of KillerRed-positive cells at each pH (Fig. 2b). In the DNA-complex-transfected cells, the fluorescence intensity and TE of the cells exhibited significantly different efficiencies of KillerRed-positive cells between the different pH values. These changes of monitoring KillerRed were imaged on a confocal microscopy (Fig. 2b). We further chose the irradiation time of 20 min for optimized ROS generation, KillerRed phototoxicity and KillerRed-mediated apoptosis for following studies (Fig. 3a,b). We also observed the ROS generated in the photoactivated KillerRed-expressed cells at pH 6.8 or 7.4 visualized with CellROX staining (Fig. 2b). In the cells treated with the DNA-complex, the slightly acidic pH had a positive effect on the ROS generation with a pronounced difference occurring between the pH values. The transgene was expressed as a function of time with a maximum intensity observed at ~48 h before subsequent decrease (Fig. 3c). The result implies the photocytotoxicity towards cells reduces over reasonable time frames, reaching ~40% of the peak concentration 7 days after transfection, and consistent with our expectations for the time frame of degradation and plasmid gene expression. Significant increase in p53 and KillerRed expression via the DNA-complex incubated at different pH values also corroborated our findings, indicating greater cellular uptake triggered with decreased environment pH.

Viability of the treated cells was determined by the 3-(4,5-dimethylthiazol-2-yl)-5-(3-carboxymethoxyphenyl)-2-(4-sulfophenyl)-2H-tetrazolium, inner salt (MTS) assay, and cells with no treatment of transfection served as a control group. According to Fig. 2c, with light irradiation alone, no significant photocytotoxicity to the control cells incubated at pH 7.4 or pH 6.8 was observed within 20 min of light exposure. Conversely, upon light irradiation, the viability of the cells treated with the DNA-complex declined markedly with a strong pH dependence, presumably due to an efficient elevation in their cellular uptake levels (Fig. 1d) and gene expression (Fig. 2a,b). Compared with the DNA-complex, the bPEI_25K_/DNA-complex-mediated cell viability showed no significant difference between pH 7.4 or 6.8 after light irradiation.

KillerRed-mediated ROS production has been reported to cause a variety of oxidative damages to lipids and cellular organs^16^, leading to an enhancement of p53-mediated apoptosis response in p53 gene-transfected cells^9^,^11^,^14^. To determine whether cell death in the photoactivated cells could be explained via the induction of apoptosis, we used the TUNEL assay to characterize apoptosis. Before light irradiation, no significant TUNEL fluorescence in the control cells and DNA-complex-treated cells was observed (Fig. 2d). Conversely, there was obvious apoptosis (~13–15%) in the bPEI_25K_/DNA-complex-transfected cells, suggesting that the effect of bPEI_25K_ toxicity has induced apoptosis^26^. However, following the light exposure treatment, an average of 67% of cells transfected with bPEI_25K_/DNA-complex at pH 7.4 were apoptotic, whereas only 15% of cells transfected with DNA-complex were apoptotic. However, this rate increased dramatically to ~70% after incubation in the slightly acidic environment (pH 6.8). These data further demonstrate that p53-mediated apoptosis here is collectively determined by pH-dependent uptake, transfection and KillerRed photoactivation.

### Genetic PDT after a single administration

**Figure 4:**
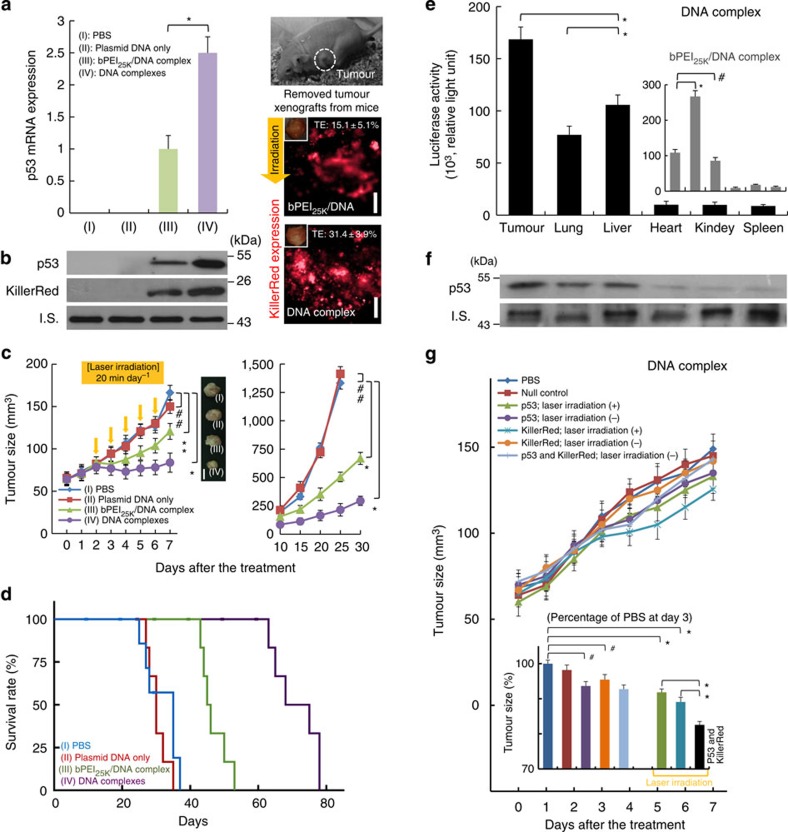
Gene expression in an aggressive H1299 mouse-tumour model after a single administration. (**a**) The p53 mRNA expression after tail vein injection; comparing PBS, plasmid DNA only, bPEI_25K_/DNA-complex and DNA-complex 48 h post injection (*P<0.00015; based on a two-tailed *t*-test, assuming unequal variances). Representative fluorescence images of excised tumour from mice 48 h after tail vein injection. Scale bar, 2 mm. The percentage of KillerRed-positive cells as TE was measured by flow cytometry. (**b**) Protein levels in tumour 2 days after tail vein injection of various complex formulations containing 10 μg pCMV-p53 and 10 μg pKillerRed-mem. Western blot analysis was performed using anti-p53 and KillerRed antibodies. β-actin protein was used as an internal standard. Total mRNA was isolated and determined for p53 by quantitative real-time reverse-transcription PCR analysis. (**c**) Effect of pH-targeting and control complexes on tumour volumes by tail vein injection. Mice were injected with various complex formulations and H1299 subcutaneous tumour volumes were measured (*P<0.00015; **P=0.013; ^##^*P*>0.1; based on a two-tailed *t*-test, assuming unequal variances). Representative samples of H1299 tumours excised on day 8 after a single treatment administration. Scale bar=5 mm. (**d**) Kaplan–Meier survival curve of mice treated with single doses by tail vein injection (**e**) Biodistribution of reporter expression after tail vein injection. Luciferase biodistribution at 48 h after injection was studied for DNA-complex or bPEI_25K_/DNA-complex containing 20 μg pCMV-luciferase (*P<0.005; ^#^*P*=0.11; based on a two-tailed *t*-test, assuming unequal variances). Inset shows the data of bPEI_25K_/DNA-complex. (**f**) Protein levels of p53 in tumour and organs 2 days after tail vein injection. (**g**) Bystander effect on H1299 subcutaneous tumour volumes by tail vein injection of various complex formulations. Inset shows a zoomed-in view of the same data. Tumour size is given as the percentage of tumour volume (mm^3^) after treatment for 3 days, compared against the PBS treatment (*P<0.005; ^#^*P*<0.05; based on a two-tailed *t*-test, assuming unequal variances). All results show mean of measurements conducted in sextuplicate±s.d.

To validate these data *in vivo* we administered carriers in an athymic BALB/c nude mouse with aggressive subcutaneous H1299 cells tumour model. Tumour excised after tail vein injection were analysed for p53 expression by PCR analysis. All the tumours from the mice injected with formulations containing bPEI_25K_/DNA-complex and DNA-complex expressed p53 while the PBS and plasmid DNA only controls did not. Remarkable expression of p53 and KillerRed was achieved when the DNA-complex was administered systemically via tail vein injection (Fig. 4a). The expression in tumour of mice 48 h after treatment with the DNA-complex measured >2 times greater compared with tumour from animals injected with the bPEI_25K_/DNA-complex. In addition, the percentage of KillerRed-positive cells as TE of DNA-complex in a single administration was over two times greater compared with bPEI_25K_/DNA-complex (Supplementary Fig. 5). Furthermore, it was equally important to validate the p53 or KillerRed expression in the tumour using immunoblot analyses. These results indicated that the pH-sensitive DNA-complex can mediate more specific and efficient gene expression (Fig. 4b). These data imply successful passive delivery of the DNA-complex to tumour site where inherent enhanced permeation and retention effect coupled with acidic microenvironment results in cellular uptake and localized, highly efficient gene transfection.

The advantages of this DNA-complex in tumour acidity-initiated plasmid DNA delivery can potentially enhance the efficiency of DNA transfection in cancer therapies within a clinical domain. Towards evaluating the therapeutic potential of the pH-triggered transfection of p53 and KillerRed, we subsequently studied tumour growth suppression after a single delivery with the bPEI_25K_/DNA-complex or DNA-complex after tail vein administration. We compared four groups of mice injected with different formulations after tail vein injection. Laser light passed by an optical fibre (Supplementary Fig. 6a) was administered to the tumour from the 2nd day for 20 min every day up to day 6 after formulation injection, relevant to the period for abatement of transgene expression following cell incubation (Fig. 3c). Body weights remained stable over the treatment periods and were similar to those of control animals (Supplementary Fig. 6b). After tail vein injection, it was observed that the DNA-complex was most effective in suppressing the established tumour volumes within a 72-h time period (Fig. 4c). As shown in other polymeric systems, tumour growth suppression stops and tumour progressively regrow after transgene p53 expression decreases^27^. No tumour growth suppression was observed when naked plasmid DNAs or PBS were administered by tail vein injection, presumably due to degradation by serum nucleases while in circulation. The median survival in animals treated with PBS, plasmid DNA or bPEI_25K_/DNA-complex was 28, 30 and 45 days, respectively (Fig. 4d). Treatment with DNA-complex increased the median survival out to 68 days, demonstrating significant antitumour activity *in vivo*. In the biodistribution of transgene expression, DNA-complex targeted gene expression in tumour achieved expression that is significantly greater than that in the lung and liver (Fig. 4e,f). When delivered *in vivo*, bPEI_25K_/DNA-complex preferentially accumulated in the lung^28^, similar to cationic liposomes^29^. In contrast, absolute expression in tumour from the DNA-complex was over 1.5 times greater than bPEI_25K_/DNA-complexes. Expression in tumour relative to lung tissue was improved by >5 fold, resulting in a dominance of specific delivery (Supplementary Fig. 7).

### Bystander effect

To assess the roles of p53 and KillerRed independently in the suppression of tumour growth, mice were tail vein injected with individual complex formulation with or without the treatment of laser irradiation (Fig. 4g). The DNA-complex containing null control did not significantly alter tumour growth. Interestingly, the DNA-complex containing KillerRed with light exposure treatment had a slight effect on the pattern of tumour size due to ROS formation by KillerRed. Data for p53 are consistent with the previous finding that p53 replenishment leads to tumour regression due to p53 protein stabilization and activation by oncogenic stress^30^. The DNA-complex containing both p53 and KillerRed conspicuously retarded tumour growth with the 20-min irradiation regimen. While DNA-complex generated a transgene expression in other organs (Fig. 4e), ROS generation is confined to the transgene expression, significantly impacting tumour tissue. bPEI_25K_/DNA-complex also achieved similar tumour suppressive activity (Supplementary Fig. 8), but the biodistribution study shows that bPEI_25K_/DNA-complex accumulated 2.5 times greater in the lung than in the tumour site (Fig. 4e, grey inset). These results suggest that bPEI_25K_/DNA-complex suffers from significant leakage and it therefore gives poor control for preferential tumour delivery compared with the DNA-complex. Although the result shows that both the mechanisms contributed to the retardation of tumour growth, the tumour growth suppression is significantly greater when p53 and KillerRed are used in conjunction with laser irradiation.

### Comparison of delivery routes

**Figure 5:**
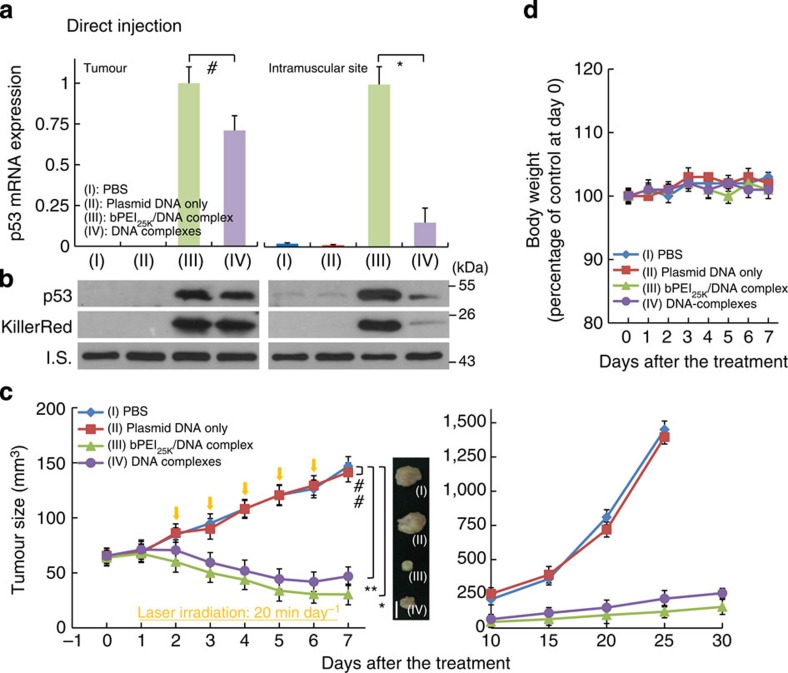
Gene expression and tumour size after a single administration by direct injection. (**a**) The p53 mRNA expression after direct injection; comparing PBS, plasmid DNA only, bPEI25K/DNA-complex and DNA-complex 48 h post injection (*P<0.00015; ^#^*P*=0.01; based on a two-tailed *t*-test, assuming unequal variances). Results show the mean of the measurements conducted in sextuplicate±s.d. (**b**) Protein levels of p53 and KillerRed in tumour 2 days after the direct injection of various complex formulations containing 10 μg pCMV-p53 and 10 μg pKillerRed-mem via the four delivery methods. Whole-tumour lysates were extracted and western blot analysis was performed using anti-p53 and KillerRed antibodies. β-actin protein was used as an internal standard. Total mRNA was isolated and determined for p53 by quantitative real-time reverse-transcription PCR analysis. (**c**) Effect of pH-targeting and control complexes on tumour volumes by direct injection. Mice were injected with various complex formulations and H1299 subcutaneous tumour volumes were measured (*P<0.00015; **P=0.013; ^##^*P*>0.1.; based on a two-tailed *t*-test, assuming unequal variances). Right panel: representative samples of H1299 tumours excised on day 8 after a single treatment administration. Scale bar, 5 mm. All results show the mean of the measurements conducted in sextuplicate±s.d. (**d**) Body weight of mice over time in response to the treatments of various complex formulations by direct injection. Results show mean of measurements conducted in sextuplicate±s.d.

We further assessed p53 and KillerRed gene expression after intratumoural and intramuscular injection to compare the delivery approach and environment pH *in vivo* (Fig. 5a,b). Intratumoural injection was evaluated for a direct comparison of the complex *in vivo.* Intratumoural versus muscular environments demonstrates an environmental response, rather than a greater response due to vascular leakiness within the tumour. No significant difference was observed between tissues for the bPEI_25K_/DNA-complex. For the DNA-complex, however, there was ~ 4.1-fold increase in gene expression of p53 in the tumour site compared with intramuscular sites. Direct injection into the tumour also had slightly improved effect over systemic delivery (Fig. 5c). Body weights remained stable over the treatment periods compared with PBS (Fig. 5d). Again, treatment with only plasmid DNAs of pCMV-p53 and pKillerRed-mem did not demonstrate any tumour growth suppression in comparison with PBS. Direct delivery of the DNA-complex or bPEI_25K_/DNA-complex both suppressed tumour growth in the murine model and significantly reduced tumour volume. The tumour inhibitory effect by intratumoural injection demonstrates that the DNA-complex is responsive to the tumoural environment, that is, the lowered pH which enables gene release, rather than being a pseudo-targeted therapy by vascular leakiness to the tumour site. Taken together, these findings convincingly demonstrate that the DNA-complex developed here effectively suppresses tumour growth through highly pH-sensitive, triggered cellular uptake and transfection of plasmid DNA in the tumour *in vivo*.

## Discussion

PDT has broad and major application and requires the use and development of sensitizing agents^17^,^31^,^32^,^33^,^34^,^35^,^36^,^37^,^38^,^39^. PDT can be applied to a large number of cancer types such as skin^31^, head and neck^32^, bladder^33^, liver cancer^34^, pancreatic cancer^35^, oesophageal cancer^36^ and prostate^17^. Further opportunities exist in preserving mucosal linings and alveoli, which suffer greatly during radiation therapy, in treatment of mouth and lung cancers, respectively. The non-ionizing nature of the radiation also allows for indiscriminate irradiation of tissues, and lesions at an early stage may be treated where surgery is preferred to be avoided or not an option. Specific to lung cancer, the U.S. Food and Drug Administration approved photodynamic therapy for the treatment of NSCLC as oncologists and surgeons seek additional tools in both its curative and palliative treatment^37^,^38^. It is important to note that therapies against NSCLC are multimodal and for advanced tumour often intend to render tumour operable for resection; such as by rendering lungs sufficient function for a patient to undergo pneumonectomy or upgraded to only requiring lobectomy. In this regard, a precise therapeutic regimen is administered to suppress growth and enable surgery. In addition, PDT for NSCLC has shown excellent palliative application significantly improving quality of life in the final months^39^. In this latter case, PDT is not applied with curative intent.

Furthermore, KillerRed can serve as a surrogate end point in regard to radiosensitization studies. Such studies can be conducted with relative ease with well-defined irradiation volumes and do not require the use of complex apparatus, dose planning and model development for irradiation experiments, for example with MeV accelerators which would struggle to deliver quantifiable, well-defined doses or shapes to a mouse model^40^.

In conclusion, a pH-responsive charge reversal polymeric complex prepared by bPEI and the biodegradable poly(PEG-His_0.5_-PEG-Glu_0.5_) copolymer, was employed to deliver both plasmid DNAs of p53 and KillerRed photosensitizer into cancer cells. We have achieved efficient transfection in tumour from systemic circulation with a switchable pH-responsive complex triggered by tumour acidosis. The DNA-complex has reversible zeta potential switching between pH 7.4 and 6.8 due to the protonation of amino groups. The unique behaviour of the DNA-complex was used to demonstrate the pH-dependent cellular uptake and transfection in the physiologically relevant pH range *in vivo*. Future development of this material could involve changing the hydrophobicity of the copolymer to load both hydrophobic and hydrophilic chemotherapeutic drugs. Furthermore, the complexes can be loaded with multiple guest molecules, reactants or drugs for dual/multiple delivery applications. Such a flexible modality adds greater potential to overcoming inter-individual variability in therapeutic response with improved specificity.

## Methods

### Materials

β-Glutamic acid hydrochloride and dimethyl sulfoxide (DMSO) were purchased from Fluka (Buchs, Switzerland). L-Histidine dihydrochloride, diethyl ether, DAPI (4′,6-diamidino-2-phenyindole, dilactate) and PBS were purchased from Sigma Co. (St Louis, MO). Poly(ethylene glycol) (PEG) diacrylate (Mn=258, density=1.11 g ml^−1^) and branched polyethyleneimine (bPEI_25K_, Mw=25,000) were purchased form Aldrich (Milwaukee, MI). Plasmid DNA of pCMV vector (pcDNA 3.1) as a null control was purchased from Invitrogen (Carlsbad, CA, USA). Plasmid DNAs of pGL3, pCMV-p53 and pKillerRed-mem were purchased from Promega (Madison, WI, USA), Clontech Laboratories (Mountain View, CA) and Evrogen JSC (Moscow, Russia), respectively. For the preparation of fluorescent-labelled plasmid DNA, pCMV-p53 and pKillerRed-mem stocks were labelled with fluorescein as a fluorescent dye using a Label IT Nucleic Acid Labeling Kit (Mirus Bio, Madison, WI).

### Synthesis and characterization of poly(PEG-His_0.5_-PEG-Glu_0.5_

Poly(PEG-His_0.5_-PEG-Glu_0.5_) was prepared according to the protocols for copolymerization (Supplementary Fig. 1)^24^. In a typical experiment using a parallel synthesizer connected to a vacuum line with the vacuum controlled by a digital vacuum controller, reaction mixtures were prepared, which contained L-histidine dihydrochloride (50.0 mg, 0.219 mmol), β-glutamic acid hydrochloride (40.2 mg, 0.219 mmol) in DMSO solvent, and the mixtures were then stirred at 25 °C for 1 h to achieve a homogeneous solution. A PEG diacrylate (101.5 μl, 0.437 mmol) was added dropwise to the mixtures, and then heated in a constant temperature oil bath at 65–75 °C for 120 h. The yellow colloidal copolymer (45% yield) was isolated and purified by washing with diethyl ether (10 ml) three times and dried at 40 °C under high vacuum (1.0 mm Hg) for 16 h.The designated functional groups and copolymer structure of poly(PEG-His_0.5_-PEG-Glu_0.5_) were confirmed by ^1^H NMR and ^13^C NMR spectra based on DMSO-_d6_ (99.8%) as a solvent^24^. The molecular weight of the poly(PEG-His_0.5_-PEG-Glu_0.5_) was determined by high-performance liquid chromatography/gel permeation chromatography analysis (HPLC/GPC, Waters model LC-2410). Tetrahydrofuran was used as an eluent in HPLC/GPC at the flow rate of 1.0 ml min^−1^. The weight- and number-average molecular weights (Mw and Mn, respectively) were calibrated using polystyrene as a reference. The poly(PEG-His_0.5_-PEG-Glu_0.5_) had a Mw of 13,000–15,000 and a polydispersity index (PDI) of 1.8–2.0. The curve of acid—base titration for the poly(PEG-His_0.5_-PEG-Glu_0.5_) was used to empirically estimate pH sensitivity and to confirm its buffering capacity from pH 10.8 to pH 3.5.

### Preparation of DNA-complexes

Test DNA-complexes at various known weight ratios (w/w/w) of bPEI_25K_/DNA/poly(PEG-His_0.5_-PEG-Glu_0.5_) and bPEI_25K_/DNA-complex were prepared by an electrostatic method in deionized water (pH 7.4; refs 24, 25). An aqueous solution of pCMV-p53 (0.5 μg) and pKillerRed-mem (0.5 μg) was mixed with an aqueous poly(PEG-His_0.5_-PEG-Glu_0.5_) solution at 1.0 μg with a final volume of 100 μl. The DNA-complexes at various known weight ratios (w/w/w) of bPEI_25K_/DNA/poly(PEG-His_0.5_-PEG-Glu_0.5_) (0.25/1/1, 0.5/1/1, 1/1/1 and 2/1/1) were obtained on addition of the mixture solution, using a pipette, into an aqueous bPEI_25K_ (0.1 μg μl^−1^) and then thoroughly mixed for 30–60 s by vortex mixing and left for at least 30 min at room temperature before measurements. In addition, the bPEI_25K_/DNA-complex at weight ratio (w/w) of 2/1 was used as a standard control.

### Characterization of DNA-complexes

The hydrodynamic diameter and the zeta potential measurements at different pH values (7.4, 7.2, 7.0, 6.8 and 6.5) were carried out using a Zetasizer Nano ZS (Malvern Instruments). The temperature for the measurement was kept at 25 °C. The concentration of the samples was 0.03% w/v. The pH value of the solutions was adjusted using 0.5 M aqueous HCl/NaOH solutions. A drop of the complex solution was allowed to air dry onto a Formvar-carbon-coated 200-mesh copper grid for transmission electron microscopy (JEOL JEM-2100F) analysis. Transmission electron microscopy images were then acquired on a JEOL-1010 microscope operating at an accelerating voltage of 100 kV.

### Cell culture and cytotoxicity of DNA-complexes

The human NSCLC cell line H1299 (CRL-5803, ATCC) was cultured in RPMI (Roswell Park Memorial Institute) 1640 medium with 10% FBS. Cells were cultured in a 37 °C incubator with 5% CO_2_. 1 × 10^5^ H1299 cells were seeded in each of the wells of a 24-well plate and fed with culture medium for 12 h. The cells were then exposed to test DNA-complexes at different weight ratios and incubated at pH 7.4 or pH 6.8 for 24 h. After 24 h incubation, the transfection media containing test samples were removed. In addition, the bPEI_25K_/DNA-complex was only incubated at pH 7.4 or pH 6.8 for 2 h and the culture medium was replaced . The CellTiter 96 AQueous one solution cell proliferation assay system (Promega, Madison, WI, USA) was used to determine the cell proliferation as per previously studies^20^,^24^,^25^. The optical density of formazan at 490 nm quantified the cell viability. The reagent contained a tetrazolium compound MTS and the reduction of MTS achieved by untreated cells was set at 100% and that of test cells was expressed as a percentage of untreated cells.

### Transfection

H1299 cells were seeded in 24-well plates at 1 × 10^5^ cells per well and transfected the next day. Culture medium was removed and cells were rinsed twice with RPMI 1640 medium without FBS (pH 7.4). Cells were replenished with 1,000 μl culture medium containing test complexes at a concentration of 1 μg plasmid DNA per well.

### Measurement of cellular uptake of DNA-complexes

To measure the cellular uptake of test complexes, we used the fluorescein-labelled plasmid DNA as the signal indicator. The measurement of cellular uptake was repeated for two cycles (30 min per cycle) of pH change between 7.4 and 6.8 when cells were transfected with test complexes. After a total of 2 h treatment, the transfection medium containing test samples was removed, the cells were rinsed twice with medium followed by analysis at different time points. In the control, the DNA-complexes or the bPEI_25K_/DNA-complex were incubated at only pH 7.4 or 6.8 for 2 h transfection. Alternatively, we evaluated the cellular uptake of test samples in the transfection medium with 10% FBS at pH 7.4 or 6.8 for 2 h transfection.

### *In vitro* assays

The fluorescence intensity of cellular uptake using the fluorescein-labelled plasmid DNA was quantitatively assessed by flow cytometry. Cells were detached by 0.025% trypsin and the suspensions were transferred to microtubes, fixed by 4% paraformaldehyde. The fluorescence intensity was determined with a flow cytometer (Beckman Coulter, Fullerton, CA, USA). Cells were appropriately gated by forward and side scatter and 10,000 events per sample were collected. The untreated cells were used as the negative control.

The p53 activities were determined in an additional 48 h post transfection. The quantitative detection of p53 protein level in cell lysates was measured by ELISA assay (Invitrogen p53 ELISA Kit, Camarillo, CA) and the absorbance of reaction reagent at 450 nm quantified the protein concentration using a spectrophotometer following the manufacturer’s directions. The detection of p53-mediated apoptosis was observed using *In Situ* Cell Death Detection Kit, Fluorescein (Roche, Mannheim, Germany) as described by manufacturer. Cells were washed with PBS and then fixed with 4% paraformaldehyde, washed twice with PBS, resuspended in permeabilization solution (0.1% Triton X-100 and 0.1% Sodium citrate) for 15 min at 37 °C and incubated with TUNEL reaction mixture for 60 min at 37 °C in a humidified atmosphere in dark. After washing three times in PBS for 2 min, the cells were analysed by a confocal microscope. The TUNEL-positive cells through p53-mediated apoptosis were further analysed using flow cytometry.

Alternatively, the transfected cells were analysed by flow cytometry (BD Bioscience, San Jose, CA, USA) equipped with a 561 nm argon laser for quantification of the fluorescence intensity and TE of KillerRed-positive cells. These cells were irradiated with yellow laser of 593 nm (100 mW cm^−2^) for various irradiation periods to quantify the amount of ROS stained with CellROX Green Reagent (Invitrogen, Camarillo, CA), whereas the control was kept in darkness. The treated cells were stained by DAPI to label the cell nuclei. The transfected cells with or without irradiation at various time periods were quantified for cell viability by MTS assay or TUNEL assay. The reduction of MTS achieved by untreated cells was set at 100% and that of test cells was expressed as a percentage of untreated cells. We further chose the irradiation time of 20 min for optimized ROS generation and KillerRed phototoxicity for following studies (Fig. 3a,b). The kinetics of p53 or KillerRed expression was investigated by examining the time course of transgene expression in cells after transfection, using spectrophotometry or flow cytometry.

### Mouse model

All procedures involving animals were permitted by Academia Sinica Institutional Animal Care and Utilization Committee (AS IACUC). Athymic BALB/c nude mice (6–8 weeks old female) were provided by National Laboratory Animal Center (Taiwan). Mice were maintained in a controlled environment with a 12 h/12 h light/dark cycle, housed in groups of five maximum and allowed food and water *ad libitum*. Cultured H1299 cells (5 × 10^6^ cells in 100 μl per mouse RPMI 1640 solution with 50% Matrigel) were injected subcutaneously into the mice. The tumour xenograft formation was monitored until reaching 5 mm in size in one plane and the volume for each individual tumour was calculated by the formula (V=(length/2) × (width)2).

### Mice study

The complex formulations (containing 10 μg pCMV-p53 and 10 μg pKillerRed-mem, 20 μg pCMV-p53, 20 μg pKillerRed-mem or 20 μg pCMV vector of null control) were injected in a volume of 100 μl via tail vein or directly into tumour of mice (athymic BALB/c nude mice with aggressive subcutaneous H1299 cells) by single injections. A yellow laser with 593 nm wavelength was used throughout this study. The light was delivered through an optical fibre and irradiated the tumour surface over a 6-mm-diameter beam spot. Animals were treated with 100 mW cm^−2^ total irradiance, determined by measuring power (PM100A, Thorlabs, Germany) before and after illumination. The tip of the laser fibre was mounted above the tumour, perpendicular to the animal. This regimen was determined following initial optimization experiments. The laser treatment was administered to the tumour for 20 min every day for 5 days starting from the 2nd day after injection. Tumour size examination was conducted 24 h after the last treatment. Survival rate after tail vein injection was examined.

### Biodistribution of reporter expression

Biodistribution of transgene expression after tail vein injection was evaluated. For examination of transgene expression *in vivo*, animals were injected with transfection complexes with 20 μg pCMV-luciferase, which was based on the luciferase gene from pGL3 cloned into pcDNA 3.1. Animals were killed at 48 h post injection. Organs were resected and homogenized in 250 mm Tris buffer, pH 7.5, using an IKA-homogenizer, frozen in liquid nitrogen and stored at −80 °C. The tissue lysates were centrifuged at 14,000*g* for 10 min at 4 °C to pelletise debris. Quantification of luciferase activity in the lysates has already been described in ref. 25. The activity of luciferase was measured with the luciferase assay system (Promega, Madison, WI, USA) according to the manufacturer’s protocols and with a 20/20n Luminometer (Turner Biosystems, Sunnyvale, CA, USA).

### *In vivo* assays

The tumour xenografts from mice injected with DNA-complexes or bPEI_25K_/DNA-complex formulations at 48 h post injection were removed and immediately stored at −80 °C. Total RNA was extracted from the homogenized tumour xenografts using RNeasy Plus Mini Kit (QIAGEN, CA, USA) and first-strand cDNA was synthesized with the Superscript III Reverse Transcriptase system (Invitrogen, CA, USA) according to the manufacturer's instructions. p53 cDNA was measured for quantitative real-time reverse-transcription PCR reaction analysis using the following sequences of primers of p53: 5′- TCTGGGACAGCCAAGTCTG -3′ and 5′- CTTCCAGTGTGATGATGGT -3′. Reporter KillerRed expression in tumour xenografts was visualized by the Nikon ECLIPSE TS100-F fluorescence microscope and reporter simultaneously excited by yellow laser through an optical fibre. Furthermore, single cell suspensions from tumour xenografts were obtained using a disaggregation procedure that resulted in efficient tumour cell recovery. In this disaggregation procedure, tumour xenografts were digested by collagenases followed by mechanical disaggregation in a BD Medimachine System (BD Bioscience, San Jose, CA, USA). Subsequently, the percentage of KillerRed-positive cells as a TE was assessed by flow cytometry.

Tumour xenograft lysates were mixed with Laemmli loading buffer, boiled, separated by sodium dodecyl sulfate polyacrylamide gel electrophoresis and transferred to a nitrocellulose membrane. Subsequently, the immunoblot analyses were performed using antibodies specific to p53 and β-actin (Thermo Fisher Scientific, IL, USA) and KillerRed (Evrogen, Moscow, Russia). The signal was developed with enhanced chemiluminescence substrate (ECL, Thermo Fisher Scientific IL, USA) after incubation with appropriate secondary antibodies. Full western blot scans are provided in Supplementary Fig. 9.

### Statistical analysis

Data are shown as the mean±s.d. for experiments performed in triplicate (*in vitro* studies) or sextuplicate (*in vivo* studies). In statistical significance testing, *P* values were calculated using a two-tailed *t*-test, assuming unequal variances. The Kaplan–Meier survival analysis was used to estimate the overall survival curve.

## Author contributions

S.-J.T., Z.-X.L., I.M.K. and P.-C.Y. designed the experiments. S.-J.T. and Z.-X.L performed the experiments of complex characterizations. S.-J.T., Z.-X.L. and Y.-F.D. carried out the *in vitro* cytotoxicity and transfection studies. S.-J.T., S.-H.K., Y.-F.Z., K.-Y.H., H.-J.L. and S.-C.Y. carried out the *in vivo* animal study. C.-L.Y. and C.-F.H. carried out the Kaplan–Meier survival curve. All authors discussed the results, co-wrote the paper and commented on the manuscript.

## Additional information

**How to cite this article:** Tseng, S.-J. *et al*. Highly specific *in vivo* gene delivery for p53-mediated apoptosis and genetic photodynamic therapies of tumour. *Nat. Commun.* 6:6456 doi: 10.1038/ncomms7456 (2015).

## Supplementary Material

Supplementary Information Supplementary Figures 1-9

## References

[b1] Hollstein M., Sidransky D., Vogelstein B. & Harris C. C. p53 mutations in human cancers. Science 253, 49–53 (1991) . 1905840 10.1126/science.1905840

[b2] Hoe K. K., Verma C. S. & Lane D. P. Drugging the p53 pathway: understanding the route to clinical efficacy. Nat. Rev. Drug Discov. 13, 217–236 (2014) . 24577402 10.1038/nrd4236

[b3] Brosh R. & Rotter V. When mutants gain new powers: news from the mutant p53 field. Nat. Rev. Cancer 9, 701–713 (2009) . 19693097 10.1038/nrc2693

[b4] Prabha S., Sharma B. & Labhasetwar V. Inhibition of tumor angiogenesis and growth by nanoparticle-mediated p53 gene therapy in mice. Cancer Gene Ther. 19, 530–537 (2012) . 22595792 10.1038/cgt.2012.26 PMC3400709

[b5] Nemunaitis J. . Biomarkers predict p53 gene therapy efficacy in recurrent squamous cell carcinoma of the head and neck. Clin. Cancer Res. 15, 7719–7725 (2009) . 19996201 10.1158/1078-0432.CCR-09-1044

[b6] Senzer N. . p53 therapy in a patient with Li-Fraumeni syndrome. Mol. Cancer Ther. 6, 1478–1482 (2007) . 17483435 10.1158/1535-7163.MCT-07-0125

[b7] Senzer N. . Phase I study of a systemically delivered p53 nanoparticle in advanced solid tumors. Mol. Ther. 21, 1096–1103 (2013) . 23609015 10.1038/mt.2013.32 PMC3666630

[b8] Wang S. P. . p53 controls cancer cell invasion by inducing the MDM2-mediated degradation of Slug. Nat. Cell Biol. 11, 694–704 (2009) . 19448627 10.1038/ncb1875

[b9] Johnson T. M., Yu Z. X., Ferrans V. J., Lowenstein R. A. & Finkel T. Reactive oxygen species are downstream mediators of p53-dependent apoptosis. Proc. Natl Acad. Sci. USA 93, 11848–11852 (1996) . 8876226 10.1073/pnas.93.21.11848 PMC38147

[b10] Seoane J., Le H. V. & Massagué J. Myc suppression of the p21(Cip1) Cdk inhibitor influences the outcome of the p53 response to DNA damage. Nature 419, 729–734 (2002) . 12384701 10.1038/nature01119

[b11] Gogna R., Madan E., Kuppusamy P. & Pati U. Reactive oxygen species-mediated p53 core-domain modifications determine apoptotic or necrotic death in cancer cells. Antioxid. Redox Signal 16, 400–412 (2012) . 22145567 10.1089/ars.2011.4103

[b12] Polyak K., Xia Y., Zweier J. L., Kinzler K. W. & Vogelstein B. A model for p53-induced apoptosis. Nature 389, 300–305 (1997) . 9305847 10.1038/38525

[b13] Rivera A. & Maxwell S. A. The p53-induced gene-6 (proline oxidase) mediates apoptosis through a calcineurin-dependent pathway. J. Biol. Chem. 280, 29346–29354 (2005) . 15914462 10.1074/jbc.M504852200

[b14] Yousefi A., Storm G., Schiffelers R. & Mastrobattista E. Trends in polymeric delivery of nucleic acids to tumors. J. Control. Release 170, 209–218 (2013) . 23770011 10.1016/j.jconrel.2013.05.040

[b15] Bulina M. E. . A genetically encoded photosensitizer. Nat. Biotechnol. 24, 95–99 (2006) . 16369538 10.1038/nbt1175

[b16] Liao Z. X., Li Y. C., Lu H. M. & Sung H. W. A genetically-encoded KillerRed protein as an intrinsically generated photosensitizer for photodynamic therapy. Biomaterials 35, 500–508 (2014) . 24112805 10.1016/j.biomaterials.2013.09.075

[b17] Moore C. M., Pendse D. & Emberton M. Photodynamic therapy for prostate cancer—a review of current status and future promise. Nat. Rev. Urol. 6, 18–30 (2009) . 10.1038/ncpuro1274 19132003

[b18] Chen J. . In vitro and in vivo gene delivery using polyethylenimine-poly(hydroxyethyl glutamine) as a non-viral carrier. J. Control. Release 152, (Suppl 1): e134–e136 (2011) . 22195803 10.1016/j.jconrel.2011.08.045

[b19] Liao Z. X. . Enhancement of efficiencies of the cellular uptake and gene silencing of chitosan/siRNA complexes via the inclusion of a negatively charged poly(gamma-glutamic acid). Biomaterials 31, 8780–8788 (2010) . 20800274 10.1016/j.biomaterials.2010.07.086

[b20] Lin C. W. . Extracellular delivery of modified oligonucleotide and superparamagnetic iron oxide nanoparticles from a degradable hydrogel triggered by tumor acidosis. Biomaterials 34, 4387–4393 (2013) . 23478033 10.1016/j.biomaterials.2013.02.058

[b21] Yoo J. W., Irvine D. J., Discher D. E. & Mitragotri S. Bio-inspired, bioengineered and biomimetic drug delivery carriers. Nat. Rev. Drug Discov. 10, 521–535 (2011) . 21720407 10.1038/nrd3499

[b22] Zeng Y. F. . Controlled delivery of recombinant adeno-associated virus serotype 2 using pH-sensitive poly(ethylene glycol)-poly-L-histidine hydrogels. Biomaterials 33, 9239–9245 (2012) . 23026709 10.1016/j.biomaterials.2012.09.018

[b23] Helmlinger G., Yuan F., Dellian M. & Jain R. K. Interstitial pH and pO2 gradients in solid tumors *in vivo*: high-resolution measurements reveal a lack of correlation. Nat. Med. 3, 177–182 (1997) . 9018236 10.1038/nm0297-177

[b24] Tseng S. J. . Switchable delivery of small interfering RNA using a negatively charged pH-responsive polyethylenimine-based polyelectrolyte complex. Chem. Commun. 49, 2670–2672 (2013) . 10.1039/c3cc00134b 23435386

[b25] Tseng S. J. . Environment acidity triggers release of recombinant adeno-associated virus serotype 2 from a tunable matrix. J. Control. Release 170, 252–258 (2013) . 23702235 10.1016/j.jconrel.2013.05.009

[b26] Moghimi S. M. . A two-stage poly(ethylenimine)-mediated cytotoxicity: implications for gene transfer/therapy. Mol. Ther. 11, 990–995 (2005) . 15922971 10.1016/j.ymthe.2005.02.010

[b27] Ndoye A. . Eradication of p53-mutated head and neck squamous cell carcinoma xenografts using nonviral p53 gene therapy and photochemical internalization. Mol. Ther. 13, 1156–1162 (2006) . 16564229 10.1016/j.ymthe.2006.02.003

[b28] Li D. . Construction of a star-shaped copolymer as a vector for FGF receptor-mediated gene delivery in vitro and in vivo. Biomacromolecules 11, 2221–2229 (2010) . 20704346 10.1021/bm100141y

[b29] Xu L. & Anchordoquy T. Drug delivery trends in clinical trials and translational medicine: challenges and opportunities in the delivery of nucleic acid-based therapeutics. J. Pharm. Sci. 100, 38–52 (2011) . 20575003 10.1002/jps.22243 PMC3303188

[b30] Ventura A. . Restoration of p53 function leads to tumour regression *in vivo*. Nature 445, 661–665 (2007) . 17251932 10.1038/nature05541

[b31] Rubin A. I., Chen E. H. & Ratner D. Basal-Cell Carcinoma. N. Engl. J. Med. 353, 2262–2269 (2005) . 16306523 10.1056/NEJMra044151

[b32] Rigual N. . Photodynamic therapy with 3-(10-hexyloxyethyl) pyropheophorbide a for cancer of the oral cavity. Clin. Cancer Res. 19, 6650–6613 (2013) . 24088736 10.1158/1078-0432.CCR-13-1735 PMC3911775

[b33] Prout G. R. . Photodynamic therapy with hematoporphyrin derivative in the treatment of superficial transitional-cell carcinoma of the bladder. N. Engl. J. Med. 317, 1251–1255 (1987) . 2959863 10.1056/NEJM198711123172003

[b34] Barathan M. . Hypericin-photodynamic therapy leads to interleukin-6 secretion by HepG2 cells and their apoptosis via recruitment of BH3 interacting-domain death agonist and caspases. Cell Death Dis. 4, e697 (2013) . 23807226 10.1038/cddis.2013.219 PMC3702308

[b35] Huggett M. T. . Phase I/II study of verteporfin photodynamic therapy in locally advanced pancreatic cancer. Br.. J. Cancer 110, 1698–1704 (2014) . 24569464 10.1038/bjc.2014.95 PMC3974098

[b36] Spechler S. J. & Souza R. F. Barrett's esophagus. N. Engl. J. Med. 371, 836–845 (2014) . 25162890 10.1056/NEJMra1314704

[b37] Akopov A. . Preoperative endobronchial photodynamic therapy improves resectability in initially irresectable (inoperable) locally advanced non small cell lung cancer. Photodiagnosis Photodyn. Ther. 11, 259–264 (2014) . 24704942 10.1016/j.pdpdt.2014.03.011

[b38] Ross P. . Incorporation of photodynamic therapy as an induction modality in non-small cell lung cancer. Lasers Surg. Med. 38, 881–889 (2006) . 17115382 10.1002/lsm.20444

[b39] Cai X. J. . Photodynamic therapy for intractable bronchial lung cancer. Photodiagnosis Photodyn. Ther. 10, 672–676 (2013) . 24284126 10.1016/j.pdpdt.2013.08.002

[b40] Begg A. C., Stewart F. A. & Vens C. Strategies to improve radiotherapy with targeted drugs. Nat. Rev. Cancer 11, 239–253 (2011) . 21430696 10.1038/nrc3007

