# Supplementary material for: Highly specific in vivo gene delivery for p53-mediated apoptosis and genetic photodynamic therapies of tumour
Source: Nat Commun. 2015 Mar 5;6:6456. doi: 10.1038/ncomms7456 (PMC4366491; doi:10.1038/ncomms7456)
Supplement: Supplementary Information — Supplementary Figures 1-9 [file ncomms7456-s1.pdf]

Supplementary Figures

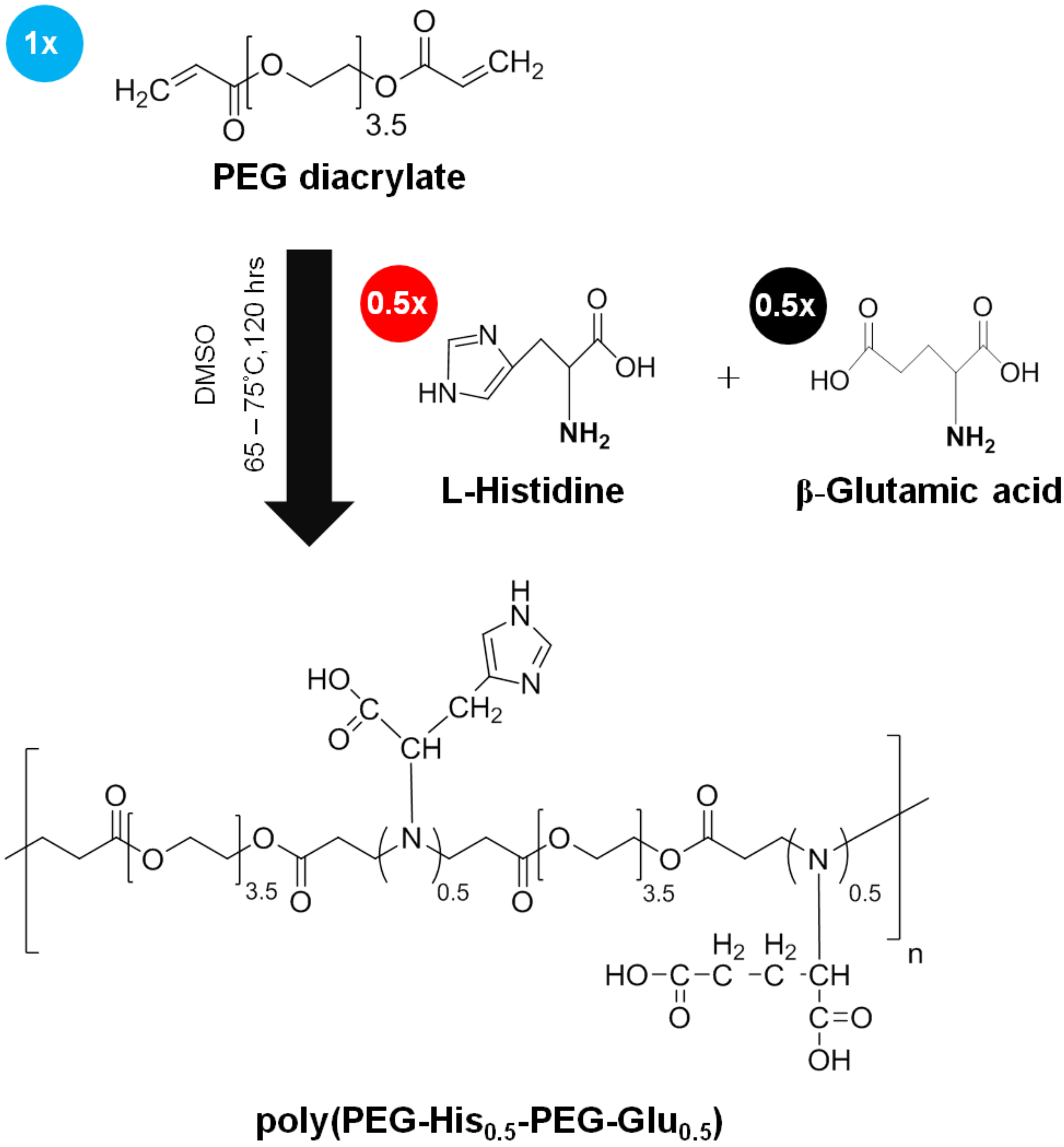

Supplementary Figure 1. Copolymer synthesis. One-stage synthesis of poly(PEG-His$_{0.5}$-PEG-Glu$_{0.5}$) via copolymerization of poly(ethylene glycol) (PEG) diacrylate with L-histidine (His), and β-glutamic acid (Glu).

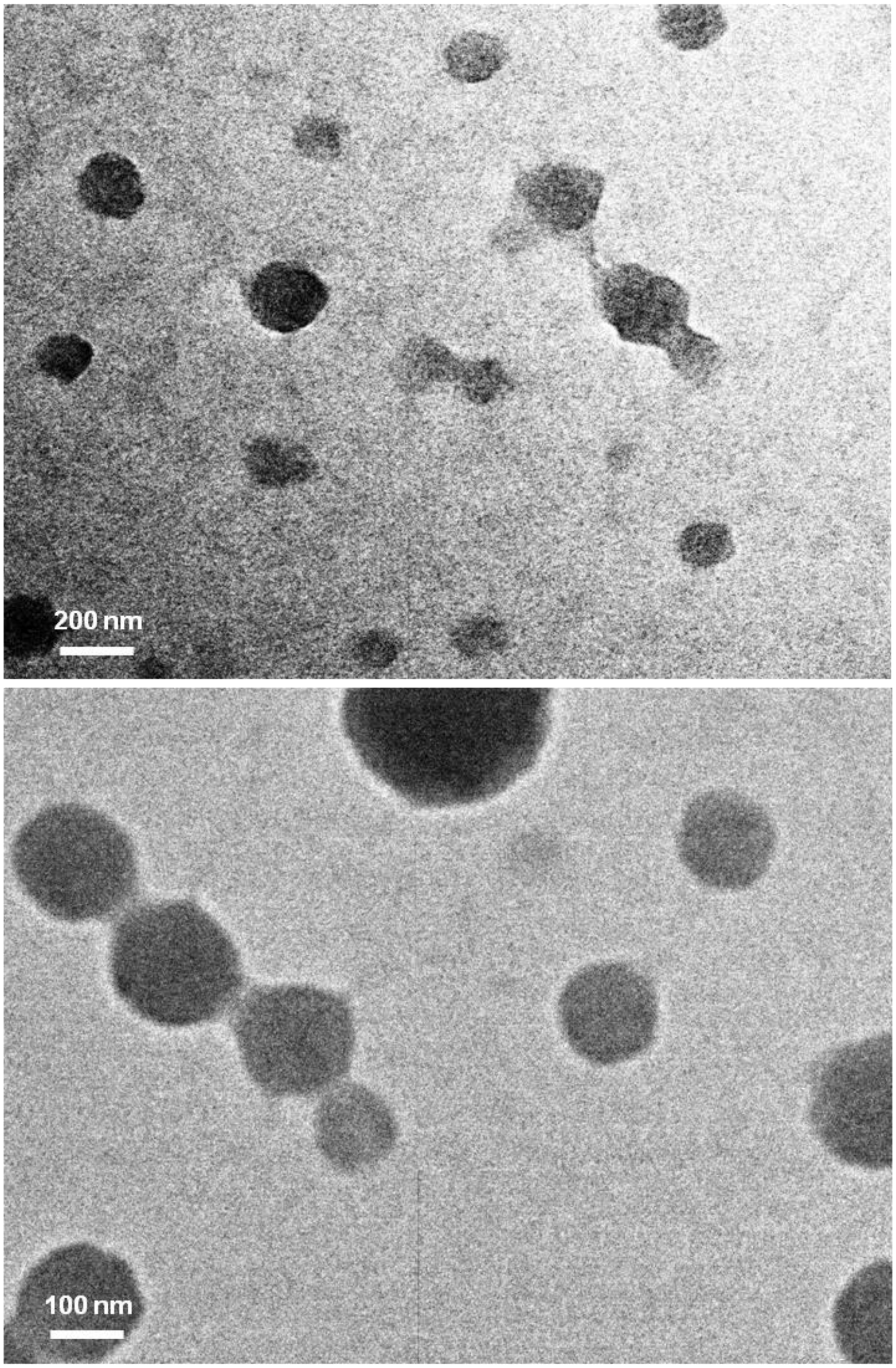

Supplementary Figure 2. TEM images of the colloidal complex. The DNA–complex at a weight ratio of 0.5/1/1) was prepared at pH 7.4. A drop of the complex solution was allowed to air-dry onto a Formvar-carbon-coated 200 mesh copper grid for TEM analysis. Bars = 100 nm.

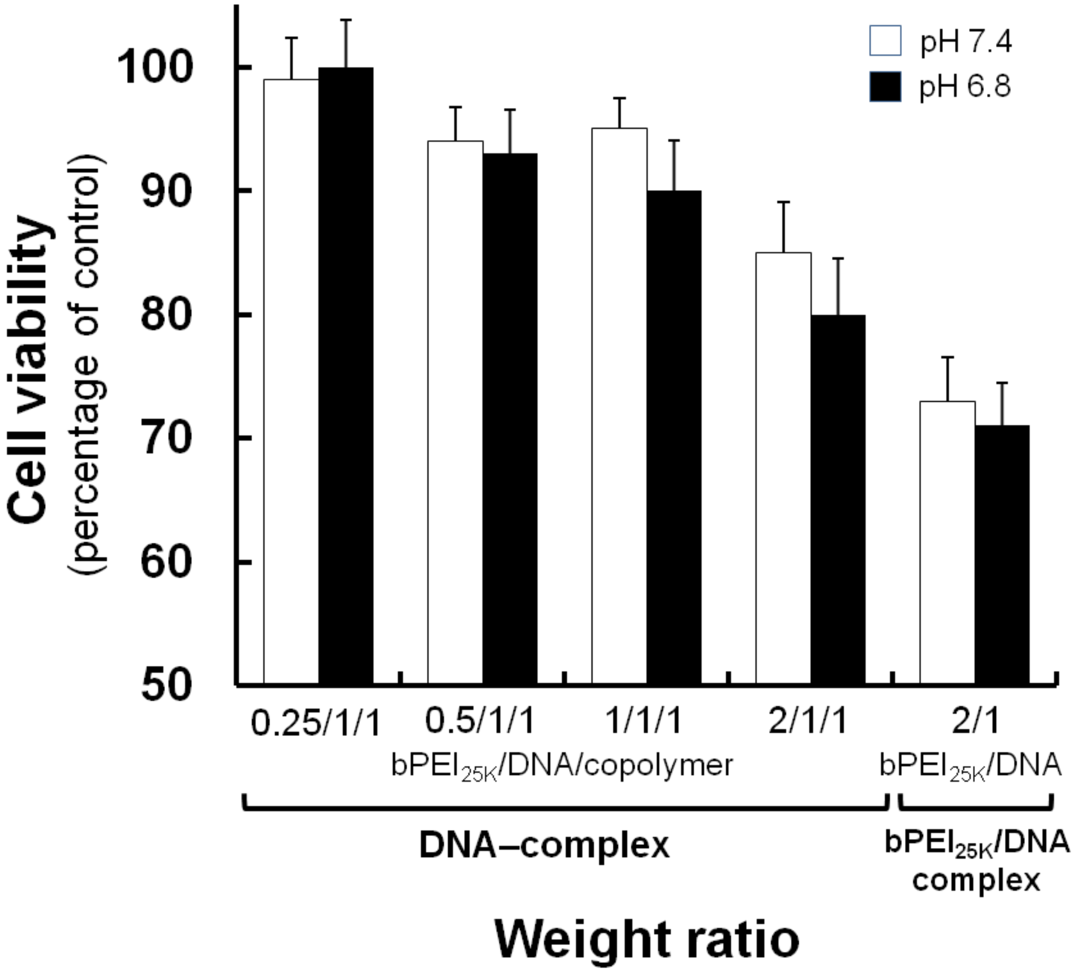

Supplementary Figure 3. Cytotoxicity of test complexes. Viability of H1299 cells after exposure to DNA–complexes at varying weight ratio (bPEI$_{25k}$/DNA/copolymer) and bPEI$_{25K}$/DNA complex at weight ratio of 2/1. Cell viability is given as the percentage of viable cells remaining after treatment for 24 h, compared against the unexposed cells. The cells were then exposed to test DNA-complexes at different weight ratios and incubated at pH 7.4 or pH 6.8 for 24 hr. The bPEI$_{25K}$/DNA complex was only incubated at pH 7.4 or pH 6.8 for 2 hr and replaced the culture medium (pH 7.4). Cell numbers were determined by the standard MTS assay. Results show mean of measurements conducted in triplicate ± s.d..

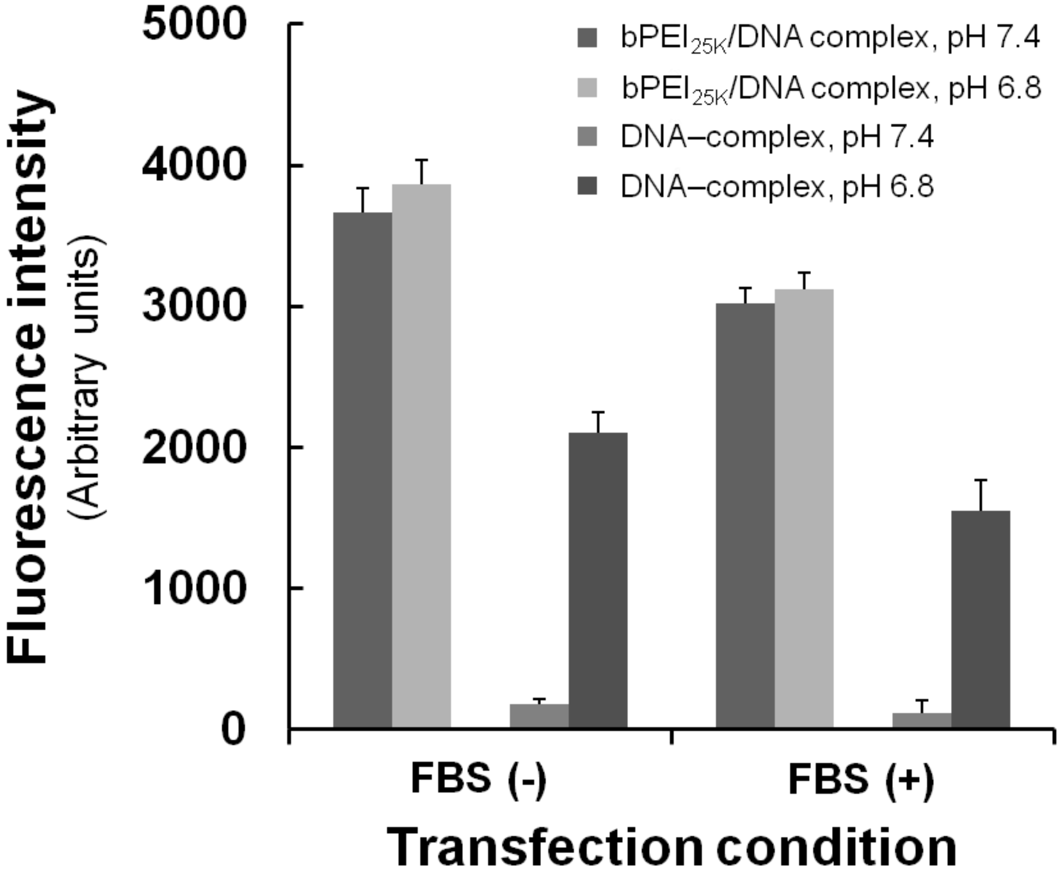

Supplementary Figure 4. Effect of serum on cellular uptake. Fluorescence intensity after cellular uptake of the $bPEI_{25K}$/DNA complex (bPEI$_{25K}$/DNA weight ratio: 2/1) or DNA–complex (bPEI$_{25K}$/DNA/copolymer weight ratio: 0.5/1/1) monitored using Alexa Fluor 488 labeling of plasmid DNAs incubated in transfection medium with or without 10% FBS at only pH 7.4 or 6.8. Results show mean of measurements conducted in triplicate ± s.d..

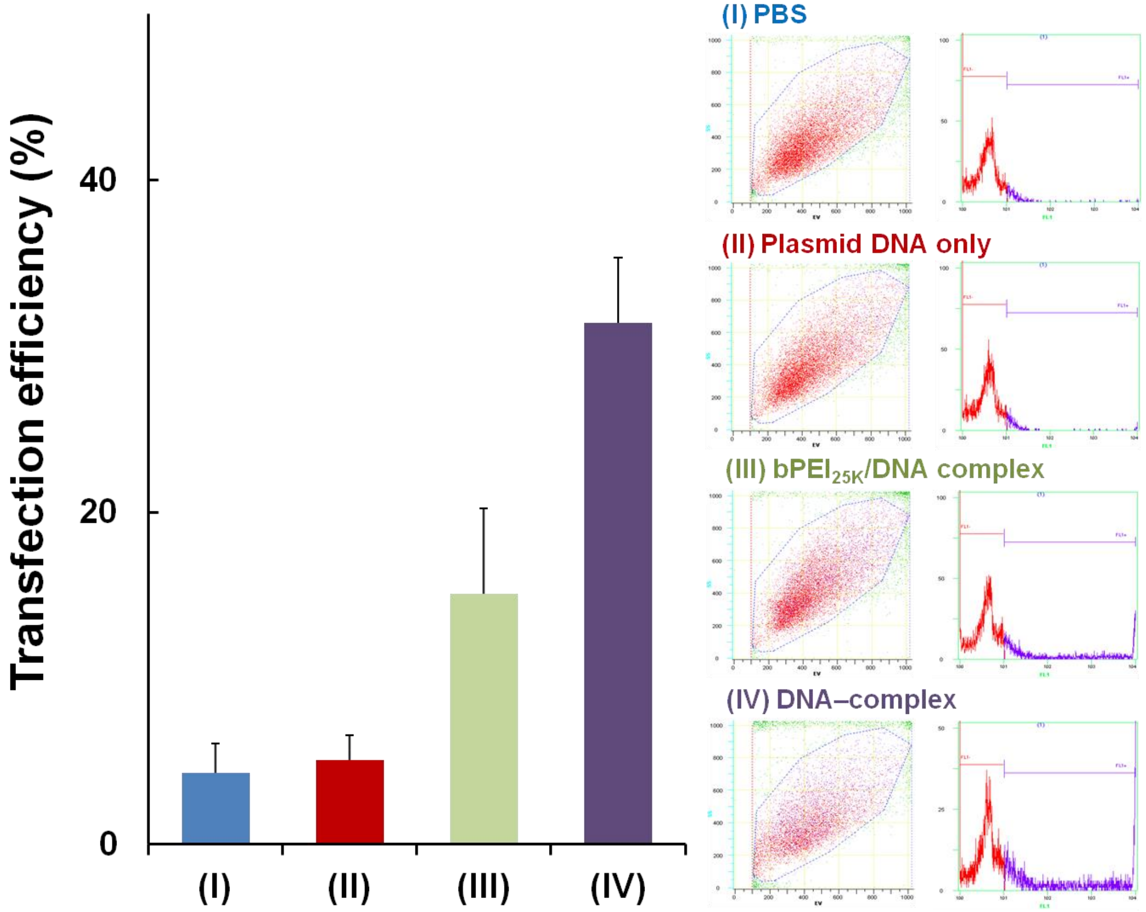

Supplementary Figure 5. Transfection efficiency in tumour by various treatments. Percentages of KillerRed-positive cells in tumour after tail-vein injection treated with PBS, plasmid DNA only, bPEI$_{25K}$/DNA complex, or DNA–complex 48 hr post-injection. Results show mean of measurements conducted in sextuplicate ± s.d..

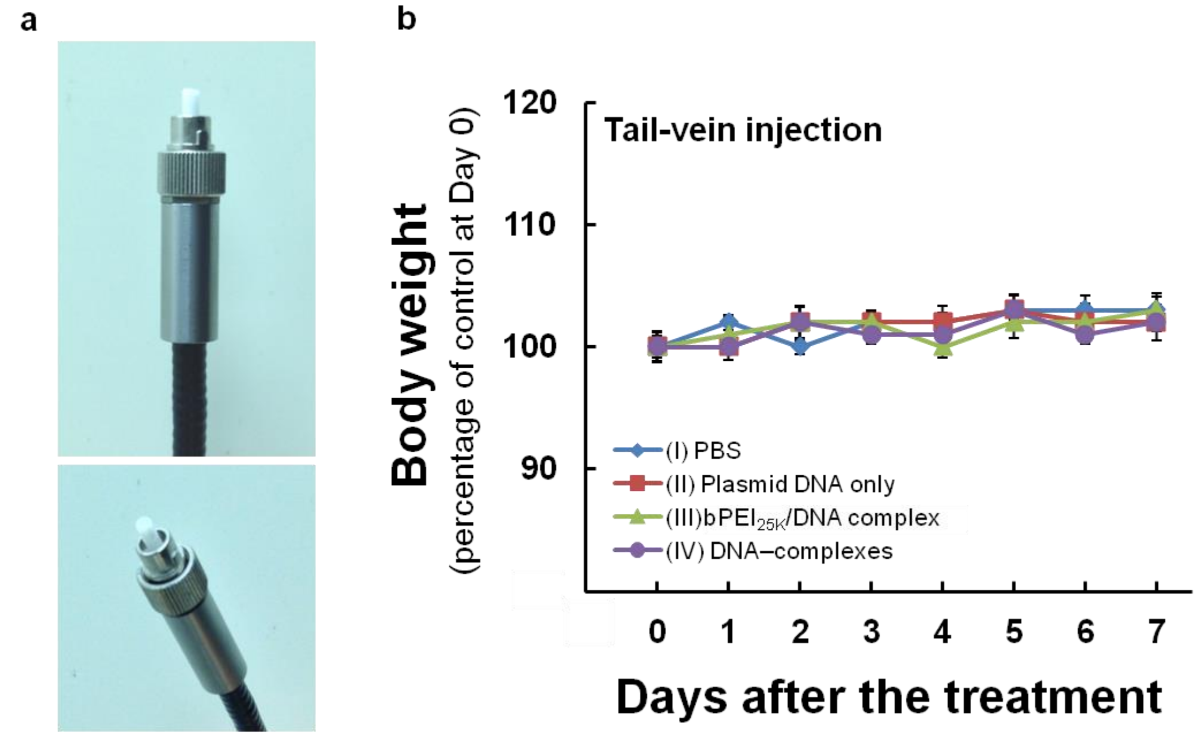

Supplementary Figure 6. Body weight of mice after various treatments. a, Photographs of the optical fiber used in mice study. **b**, Body weight of mice over time in response to the treatments of various complex formulations by tail-vein injection. Results show mean of measurements conducted in sextuplicate ± s.d..

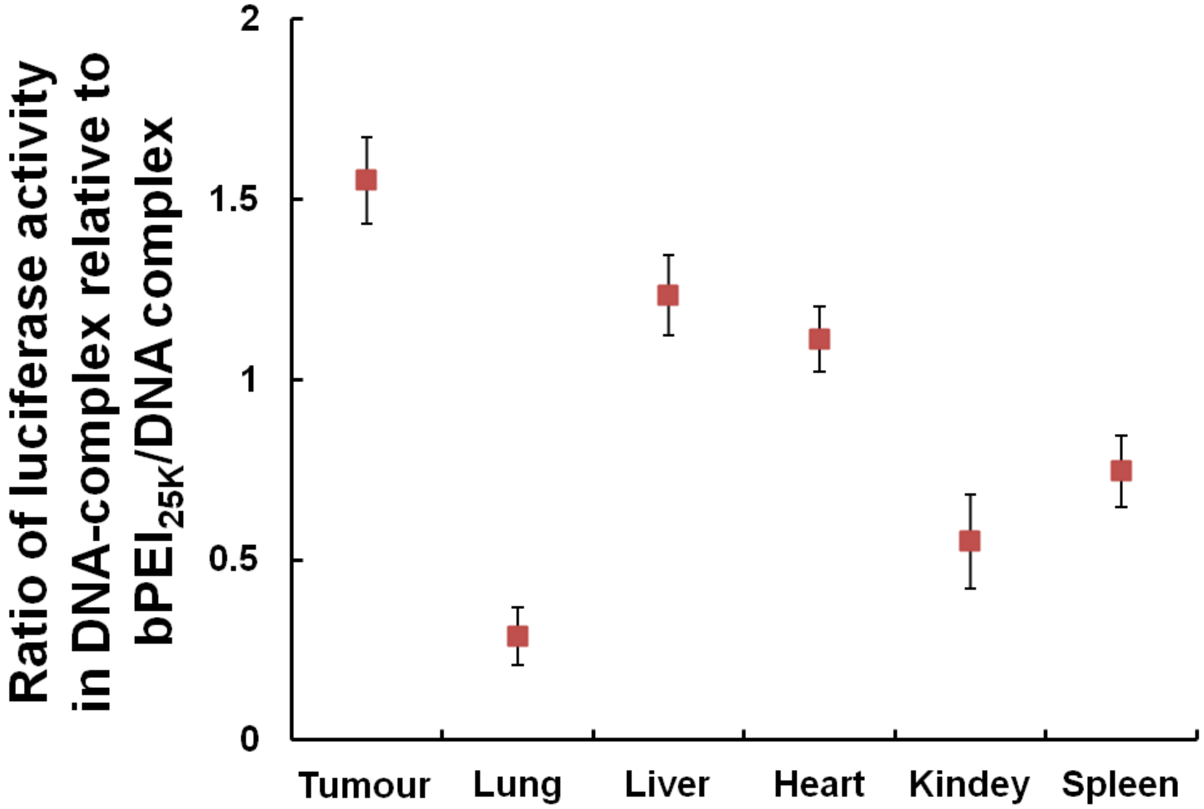

Supplementary Figure 7. Biodistribution ratio of transgene expression by pH-sensitive complex. Comparison of the ratio of luciferase expression from DNA–complex delivery relative to $bPEI_{25K}$/DNA complex. Results show mean of measurements conducted in sextuplicate ± s.d.

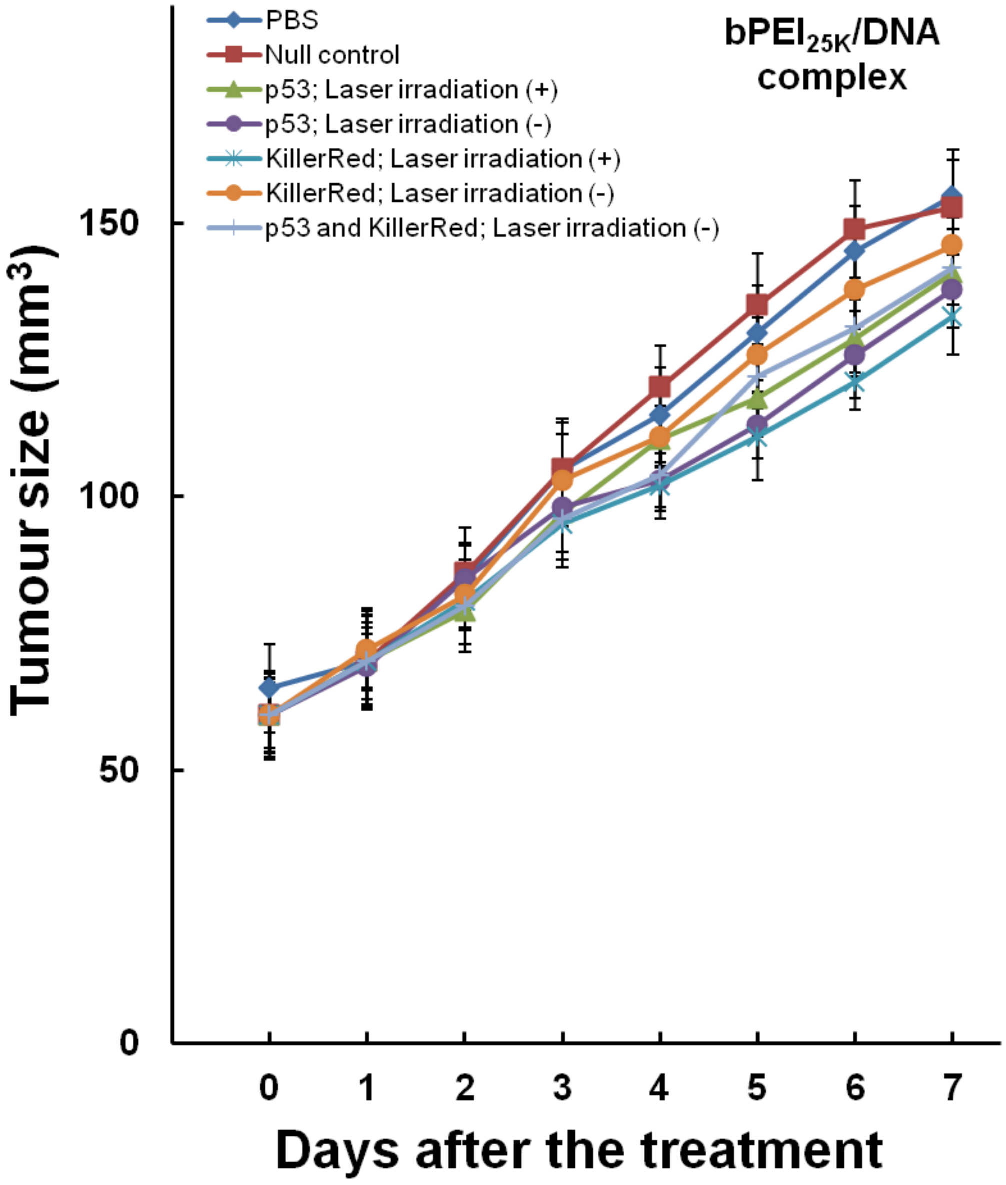

Supplementary Figure 8. Gene expression after a single administration of various bPEI$_{25K}$/DNA complex formulations. Effect of control complexes on tumour volumes by tail-vein injection. Mice were injected with various complex formulations and H1299 subcutaneous tumour volumes were measured. All results show mean of measurements conducted in sextuplicate ± s.d.

Fig 4.

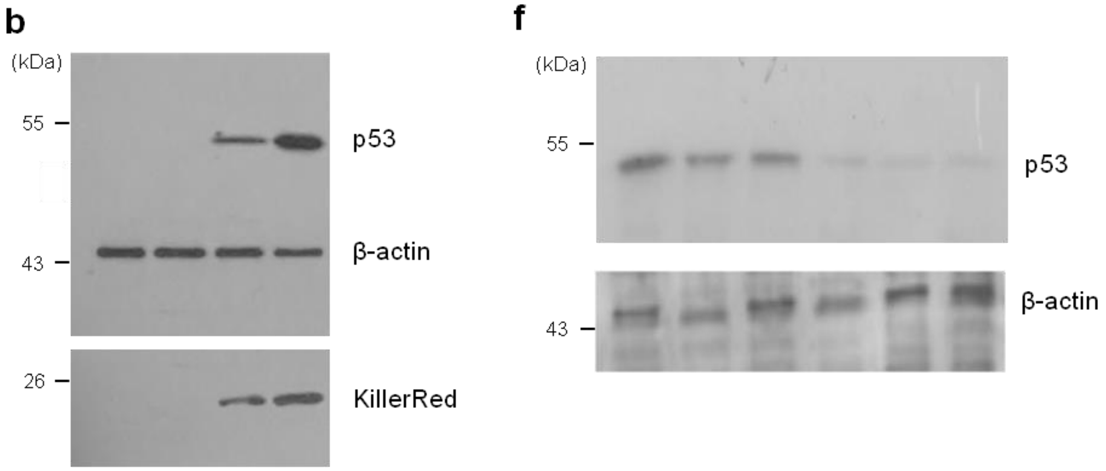

Fig 5.

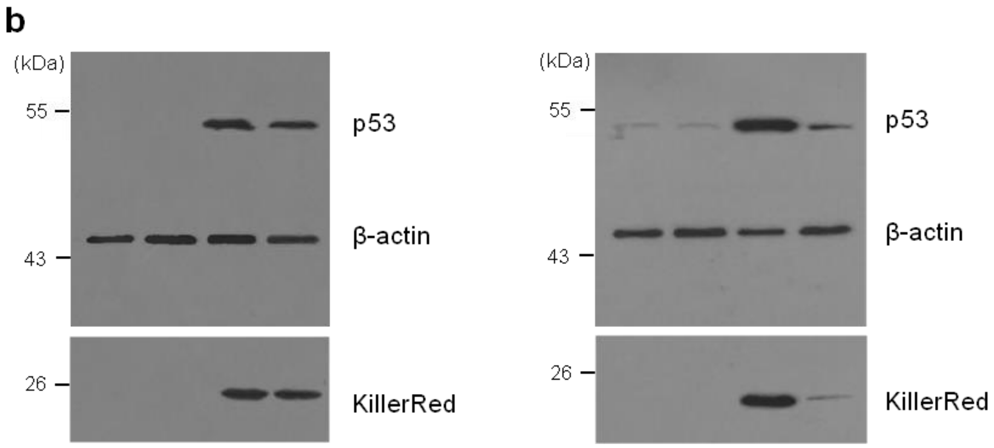

Supplementary Figure 9. Full scans of key Western blot data shown in Fig 4b, 4f, and 5b.
